# Sleeplessness

**Published:** 1897-10

**Authors:** 


### Abstracts and Selections.

### Sleeplessness.

It is unfortunate that the physiology of sleep is not better understood. It is even more unfortunate that what we do know about the phenomenon of sleep is not more diffused among the profession. How frequently indeed, do we observe physicians who mistake unconsciousness for sleep, and who seemingly regard the goal of the application of their therapeutical measures, as having been reached, when they have succeeded in rendering a tired, worn out, nervous individual oblivious to his surroundings by placing him in a condition which they call sleep, but which is, simply unconsciousness. An experience of several years in insane hospital practice presented to the writer, in a very strong light, the enormity of this error and its wide prevalence. It was no uncommon sight, to see patients brought to the hospital in an unconscious state, due to the administration of narcotics; opium or morphine being the favorite drug used. One case is worthy of mention here, a male, neurasthenic, harmless, but suffering from the exaggerated mental symptoms, so marked in this disease, was accompanied by his physician to the hospital. The physician stated his reason for coming was, that he considered it important to keep his patient asleep, so that he would not appreciate the humiliation of entering the hospital, and further, that inasmuch as he had not been sleeping well for some time, and that he was now sleeping, he considered the opportunity a good one, to let him recuperate. To this end, the physician was at intervals giving hypodermic injections of morphine, and the poor, worn-out, narcotized patient, saturated with morphine, and believed to be asleep, was, as best he could, with his worn-out nervous mechanism, fighting for his life, which his respiration, weak pulse and inhibited reflexes, showed was at very low ebb. This case was not an exceptional one; it was, alas, too common an experience. The physician had lost sight of the very essential principle which should govern our therapy in the treatment of sleeplessness, and that is, that sleep has for its object the repair of the wear and tear of vital processes of life, and to insure sleep we must not interfere with these processes, which we do when drugs are given until sedation results.

There is another thing to bear in mind in the consideration of sleeplessness, and that is, that there is a source of irritation somewhere in the economy, which, if relieved, will be followed by sleep. Again, "an axiom" well worth remembering, is, that the more gentle the means employed to induce sleep, the more natural will be the sleep induced, and the more gentle the means employed, the more careful must we be to select the right time for their use. We believe that Dujardin-Beaumetz was right when speaking of the use of drugs in the treatment of sleeplessness; he said: "That for a drug to be hypnotic, it must imitate the natural condition of sleep, by effecting a lowered intracranial pressure and that drugs which, though bringing about unconsciousness do not lower cerebral pressure, or which increase it, cannot claim to be hypnotics. Opium and morphine are objectionable on this ground. Drug treatment must put the patient in a position to go to sleep in a natural way, and not *put* him to sleep." Sedatives do this, but narcotics do not.

In the use of sedatives we must be cautious and not use them *ad libitum*. The writer in a paper on "Sedatives in the Treatment of Insanity" (1892, *Hospital Bulletin of Minnesota*), said, "Each case is a 'law unto itself,' and as such requires patient and persistent study ere we commit the folly of giving a hypnotic, when more simple and efficacious methods would produce satisfactory results." You cannot cure sleeplessness by drug treatment; the drugs simply conserve nervous energy and act as valuable assistants to the building-up process, necessary to cure the sleeplessness. Sedatives act, as before stated, by placing the patient in a position to go to sleep, and nature does the rest.

In our experience we have learned to rely upon the bromides, chloral, cannabis indica and hyoscyamus as sedatives, which, if judiciously used, brings order out of chaos. The bromides lower the sensibility of the brain, and thus promote sleep. The single salts can be used, but in the writer's experience, where a sedative is indicated in sleeplessness, it is better to combine them, and when there is any excitement, add chloral. Cannabis indica is a sedative which is but little used by the general practitioner, and for the reason that it is misunderstood, misrepresented, and as a result never used as it should be. Clouston, Mathison and Echeverria have taught us their value. Hyoscyamus is another sedative, the value of which is not appreciated, a drug which is endorsed by Budde, Brush, Krafft-Ebing as a hypnotic. Now, these valuable sedatives, when combined, give us a thoroughly reliable and satisfactory agent, with which to treat sleeplessness, and in the writer's experience no more elegant or reliable preparation is before the profession than that of Bromidia, in which is combined in proper proportion, the bromide of potassium, chloral hydrate, hyoscyamus and cannabis indica. We feel that the profession can always rely upon this combination, and find it especially useful in the treatment of sleeplessnes.—*The Medical Fortnightly.*

